# Factors predicting statin prescribing for primary prevention: a historical cohort study

**DOI:** 10.3399/bjgp20X714065

**Published:** 2021-02-09

**Authors:** Samuel Finnikin, Brian H Willis, Ronan Ryan, Tim Evans, Tom Marshall

**Affiliations:** Institute of Applied Health Research, University of Birmingham, Birmingham.; Institute of Applied Health Research, University of Birmingham, Birmingham.; Public Health England, London.; Public Health England, London.; Institute of Applied Health Research, University of Birmingham, Birmingham.

**Keywords:** cardiovascular diseases, cholesterol, decision making, shared, general practice, hydroxymethylglutaryl-CoA reductase inhibitors, risk assessment

## Abstract

**Background:**

Initiation of statins for the primary prevention of cardiovascular disease (CVD) should be based on CVD risk estimates, but their use is suboptimal.

**Aim:**

To investigate the factors influencing statin prescribing when clinicians code and do not code estimated CVD risk (QRISK2).

**Design and setting:**

A historical cohort of patients who had lipid tests in a database (IQVIA Medical Research Data) of UK primary care records.

**Method:**

The cohort comprised 686 560 entries (lipid test results) between 2012 and 2016 from 383 416 statin-naive patients without previous CVD. Coded QRISK2 scores were extracted, with variables used in calculating QRISK2 and factors that might influence statin prescribing. If a QRISK2 score was not coded, it was calculated post hoc. The outcome was initiation of a statin within 60 days of the lipid test result.

**Results:**

Of the entries, 146 693 (21.4%) had a coded QRISK2 score. Statins were initiated in 6.6% (95% confidence interval [CI] = 6.4% to 6.7%) of those with coded and 4.1% (95% CI = 4.0% to 4.1%) of uncoded QRISK2 (*P*<0.001). Statin initiations were consistent with National Institute for Health and Care Excellence guideline recommendations in 85.0% (95% CI = 84.2% to 85.8%) of coded and 44.2% (95% CI = 43.5% to 44.9%) of uncoded QRISK2 groups (*P*<0.001). When coded, QRISK2 score was the main predictor of statin initiation, but total cholesterol was the main predictor when a QRISK2 score was not coded.

**Conclusion:**

When a QRISK2 score is coded, prescribing is more consistent with guidelines. With no QRISK2 score, prescribing is mainly based on total cholesterol. Using QRISK2 is associated with statin prescribing that is more likely to benefit patients. Promoting the routine CVD risk estimation is essential to optimise decision making.

## INTRODUCTION

Statins are the most prescribed class of medicine in England^1^ with >70 million statin prescriptions issued each year,^2^ and they play an important role in the primary prevention of cardiovascular disease (CVD). The predicted benefit of taking statins is proportional to the estimated risk of CVD,^3^ but the potential harms and costs are independent of CVD risk. Therefore, there is a risk threshold where the predicted benefits outweigh the harms, and statins can be recommended. Because of this, the estimation of CVD risk is a fundamental part of clinical guidance on CVD prevention around the world.^4^^–^^7^ In England and Wales, the risk threshold for offering treatment was a 20% 10-year risk until 2014, when it was lowered to a 10% 10-year risk.^7^^,^^8^ The effective utilisation of risk scoring improves accuracy of CVD risk predictions and increases medical prescribing with no evidence of clinical harm.^9^^–^^11^ Moreover, because communication of the risks and benefits of treatment options is a necessary component of shared decision making, an accurate CVD risk estimate is an essential part of effective clinical decision making.

However, patients are often initiated on statins when their estimated CVD risk is below the recommended threshold,^12^^–^^16^ suggesting that prescribing decisions are not wholly based on risk estimates. One reason for this is a focus on individual risk factors: the *‘heuristic that identifies elevated cholesterol as a medical problem in its own right’*.^17^ One US study showed single risk factor management strongly influenced statin prescribing,^18^ and a randomised theoretical experiment in Australia highlighted clinicians’ preferences for managing individual risk factors over absolute risk.^19^ This preference is consistent with observational data, which showed that statin initiation in Australia was largely unrelated to CVD risk, whereas in New Zealand it was more aligned to CVD risk.^20^ Other reasons that clinicians may not use risk scores include a belief that risk scoring oversimplifies the decision and may lead to overprescribing^21^ and confusion about how best to use risk score calculators.^22^ Finally, patients find it difficult to make decisions based on future risks and tend to preferentially focus on cholesterol levels when considering taking statins,^23^^,^^24^ which is likely to influence clinical decision making.

This study explores the prescribing of statins for the primary prevention of CVD in England and Wales. Previous research^16^ has shown low rates of coding of estimated CVD risk (QRISK2),^25^ so this study utilises post-hoc risk estimates to allow for the possibility that clinicians calculate, but do not code, estimated CVD risk, nor estimate CVD risk using clinical judgement. This also enables investigation of whether the act of coding estimated CVD risk is associated with prescribing informed by CVD risk. By focusing on untreated patients with a coded lipid result, the period of CVD risk assessment and potential consideration of statin treatment is observed. This enables a greater understanding of the way GPs make decisions in practice and how risk scoring influences prescribing decisions. The objective of this study, therefore, is to identify the factors influencing statin prescribing when clinicians code, and do not code, estimated cardiovascular disease risk.

**Table:** How this fits in

Estimated cardiovascular disease risk provides the best guide to the benefits of using statins for primary prevention. However, it is not always carried out prior to initiation of statins, which brings into question how statin initiation decisions are made. By focusing on the period of time following a lipid test, it was found that, when cardiovascular risk is calculated and coded, the prescribing decision-making process is clearly different from when cardiovascular risk is not coded. When risk estimates are coded, statin prescribing is much more aligned with clinical guidance. But, when risk estimates are not coded, prescribing is mainly influenced by total cholesterol levels and therefore more discordant with guidance.

## METHOD

This was a historical cohort study using data from an anonymised database of primary care records for practices in England and Wales contributing to IQVIA Medical Research Data (IMRD)-UK (formally The Health Improvement Network). IMRD contains routine patient data from >500 general practices, and is generalisable to the UK population.^26^ Practices contributing to IMRD use the Vision (In Practice Systems) electronic patient records system.^27^ Clinical classification version 2 Read codes are used for clinical data^28^ and *British National Formulary* (BNF) drug codes for prescribing data.^29^

To maintain a stable cohort of practices, only IMRD practices contributing data for the whole study period from the beginning of 2012 to the end of 2016 were included. Patients registered in an IMRD practice, aged 40–85 years, with a lipid result, and without a previous statin prescription were eligible for inclusion. The lower age cut-off was chosen to correspond with the start of NHS Health Check eligibility, and the upper limit was chosen to correspond to the maximum age where QRISK2 can be used. Exclusion criteria included existing CVD, familial hypercholesterolaemia, and pregnancy (see Box 1 for full list). Patients without 60 days of follow-up were also excluded. An individual patient with multiple lipid results may enter the cohort multiple times. The unit of analysis is an entry (an occasion when statins might be prescribed) rather than an individual patient.

**Box 1.** Cohort inclusion and exclusion criteria

**Inclusion criteria**
Age 40–85 years
Lipid result coded (at least TC and HDL) in study period
No previous statin prescriptions

**Exclusion criteria**
Existing CVD
Chronic kidney disease stage ≥3
Type 1 diabetes mellitus
Hypercholesterolaemia
Pregnancy
Incomplete follow-up (60 days)

**Dates defining eligibility**
Latest of:
Study start date
Acceptable mortality reporting date
Vision installation date plus 1 year
Registration date plus 1 year
40^th^ birthday
Until earliest of:
85^th^ birthday
Study end date
New CVD diagnosis
New statin initiation
New recording of a contraindication to the prescribing of statins
Death
Transfer out of the practice

CVD = cardiovascular disease. HDL = high-density lipoprotein. TC = total cholesterol.

Demographic data on practice identifier, ethnicity, Townsend deprivation quintile,^30^ sex, and age were extracted. For missing data a ‘missing’ category was used. The lipid values were extracted, along with any variables that are included in the QRISK2 calculation or could influence statin prescribing (see protocol paper^31^ for full list). Biologically implausible values were excluded (including cases where high-density lipoprotein [HDL] cholesterol was greater than total cholesterol [TC]).

Any QRISK2 score coded from the date of the lipid result to 60 days later was extracted. The follow-up period was 60 days from the index date (the date of the lipid result being coded, or the date of QRISK2 score coding). The follow-up period was chosen as a time period during which it would be considered clinically reasonable that the decision to start a statin was related to the lipid measurement or the coded QRISK2 score. The date of QRISK2 coding was used as this demonstrates clinical interaction with the lipid result, and hence can be taken as a point where management decisions are made. In the absence of a coded QRISK2 score, it was assumed that the clinician filing the lipid result would act on this information at this time.

### Analysis

The dataset was divided into two groups: cohort entries with a QRISK2 score coded 0–60 days after the lipid result, and those without. A post-hoc QRISK2 score was calculated for each entry into the cohort using their risk factor data. Where variables were missing, the QRISK2 calculator default values were used in the same way as they would in clinical practice.

The cohort was described, including mean lipid values, QRISK2 scores, and the proportion of entries resulting in a statin prescription. The coded or post-hoc calculated QRISK2 scores were used to assess if statin initiation was indicated according to National Institute for Health and Care Excellence (NICE) guidelines (10-year QRISK2 score ≥20% pre-2014 and ≥10% post-2014). The proportion of indicated and non-indicated statin initiations were calculated for those with and without a coded QRISK2 score. Because changes to guidelines were announced prior to their publication, and to allow for a dissemination period after their publication, data from 2014 were excluded in this part of the analysis as it is difficult to define an exact date on which general practices transitioned from one guideline to the other.

A multivariable logistic regression model was fitted to model statin initiation. The initial full model included all the covariates in the QRISK2 score, the calendar year, and TC. All continuous covariates were included as multiple fractional polynomials in order to establish their functional form.^32^ Discrete Bernoulli or ordinal variables were included in their original form. The outcome was the prescription of a statin within 60 days.

There were three steps to the elimination of covariates to derive the eventual model:

1. Backward elimination was used to remove insignificant covariates (*P*>0.05) to produce an initial fixed-effects model.
2. A random intercept for the practice identifier (ID) was then introduced and insignificant variables were identified and eliminated.
3. If no insignificant variables were identified at step 2, the resulting mixed-effects model was the reduced model adopted.

If insignificant covariates were identified then steps 1 and 2 were repeated until there were no insignificant variables at step 2. The resulting model was described for the coded and uncoded groups. To indicate the discriminative and predictive ability of the models, receiver operating characteristic (ROC) curves and calibration plots (using LOESS smoothing) were derived.^33^

Finally, the prescribing pattern of the coded group was applied to the uncoded group to estimate the rates of statin prescribing if the uncoded group behaved in the same way as the coded group. All statistical analyses were conducted in R (version 3.5.2).

## RESULTS

**Figure 1.**
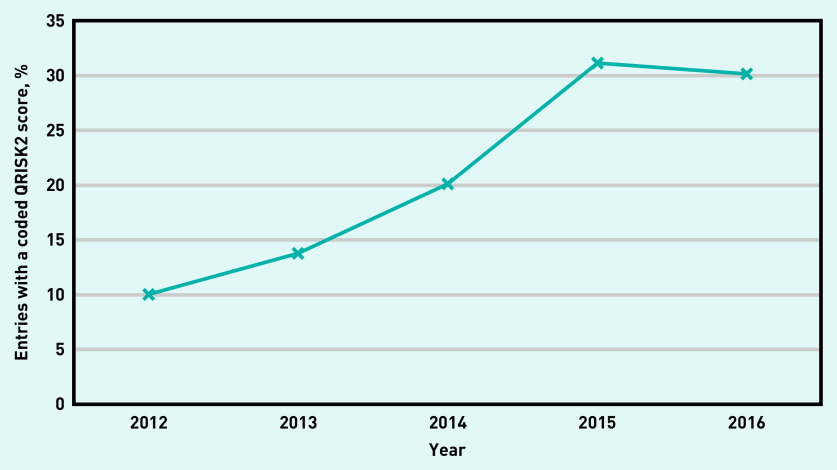
***Percentage of entries with a coded QRISK2 score by year.***

There were 686 560 entries into the cohort from 383 416 individual patients and 227 separate practices. Of these, 146 693 entries (21.4%; 95% confidence interval [CI] = 21.3% to 21.5%) (*n* = 128 009 individual patients) had a coded QRISK2 score 0–60 days after coding of the lipid result (see Supplementary Table S1 for details). The proportion of entries where a QRISK2 score was coded increased over the study period but remained well below one-third (Figure 1). The groups were broadly similar, although there were higher levels of comorbidity, obesity, and older patients in the uncoded group (see Supplementary Tables S1 and S2). The higher mean QRISK2 score in the uncoded group is largely due to the differences in age between the two groups, with 10.3% of the coded group being ≥70 years old compared with 16.3% of the uncoded group. There were also significantly fewer lipid tests per patient in the coded versus uncoded groups (1.15 versus 2.11 tests per patient; *P*<0.01).

There was good agreement between calculated and recorded QRISK2 scores (*R*^2^ = 0.874). Overall, a statin was initiated within 60 days of the index date for 4.6% (95% CI = 4.6% to 4.7%) of entries, and 30.5% (95% CI = 30.0% to 31.0%) of these initiations had a coded QRISK2 score. Statin initiations were more common among the entries with a coded QRISK2 score (6.6%; 95% CI = 6.4% to 6.7%) compared with 4.1% (95% CI = 4.0% to 4.1%) in the uncoded group (*P*<0.001) (see Supplementary Table S1).

Entries in the coded group were more likely to be prescribed a statin when it was indicated than the uncoded group (risk ratio [RR] = 3.11, 95% CI = 3.02 to 3.21) (Table 1). In the uncoded group, 44.2% (95% CI = 43.5% to 44.9%) of statin prescriptions were indicated in accordance with NICE criteria compared with 85.0% (95% CI = 84.2% to 85.8%) of statin prescriptions in the coded group (RR = 1.92, 95% CI = 1.89 to 1.96). In both groups, the proportion of entries prescribed a statin when it was indicated was low (6.2% when QRISK2 was not coded, 19.2% when it was) (Table 1).

**Table 1.** Rates of initiation according to whether statins would be indicated according to prevailing guideline

**Group^a^**	**Indication**	**Statin initiated, *n* (%)**	**Statin not initiated, *n* (%)**	**Total, *n***
Uncoded	Indicated	7550 (6.2)	114 471 (93.8)	122 021
	Not indicated	9532 (3.2)	291 448 (96.8)	300 980
	Total	17 082 (4.0)	405 919 (96.0)	423 001

Coded (indication by calculated QRISK2)	Indicated	6252 (19.2)	26 243 (80.8)	32 495
	Not indicated	1959 (2.3)	82 821 (97.7)	84 780
	Total	8211 (7.0)	109 064 (93.0)	117 275

Coded (indication by coded QRISK2)	Indicated	6978 (19.2)	29 286 (80.8)	36 264
	Not indicated	1233 (1.5)	79 778 (98.5)	81 011
	Total	8211 (7.0)	109 064 (93.0)	117 275

a Excluding the 146 284 entries from 2014.

In the regression analysis for entries with a coded QRISK2 score, the QRISK2 score was the most important variable in explaining the variation in statin prescribing (see Supplementary Table S3 for details), followed by TC. The other contributing variables (in order of contribution) were type 2 diabetes, TC/HDL ratio, hypertension, year, rheumatoid arthritis, Townsend deprivation quintile, smoking status, and sex. In the regression model for entries without a coded QRISK2 score, TC was by far the most important predictor, followed by type 2 diabetes and then (post-hoc) QRISK2 score. TC/HDL ratio, sex, age, body mass index (BMI), hypertension, smoking status, atrial fibrillation, Townsend deprivation quintile, and family history of CVD also contributed with diminishing importance. Both models showed good discrimination and calibration (see Supplementary Figure S1 for details) with area under curves (AUC) of 0.864 (95% CI = 0.861 to 0.868) and 0.838 (95% CI = 0.834 to 0.842), for the coded and uncoded models, respectively. Calibration of the models is poorer at higher predicted probabilities of statin prescribing owing to the low number of entries with these predicted probabilities.

Using the model created for the coded group, the distributions of predicted probabilities (of statin initiations) for the two groups are very similar (see Supplementary Figure S2 for details). Therefore, it is possible to estimate what the effects on statin prescribing would be if GPs had coded QRISK2 scores in the uncoded group. If this were the case, the number of statin initiations (excluding 2014) would increase from 17 082 (4.0%) to 27 943 (6.6%) and the proportion of prescriptions that are consistent with NICE guidelines would increase from 44.2% to 83.8%.

**Figure 2.**
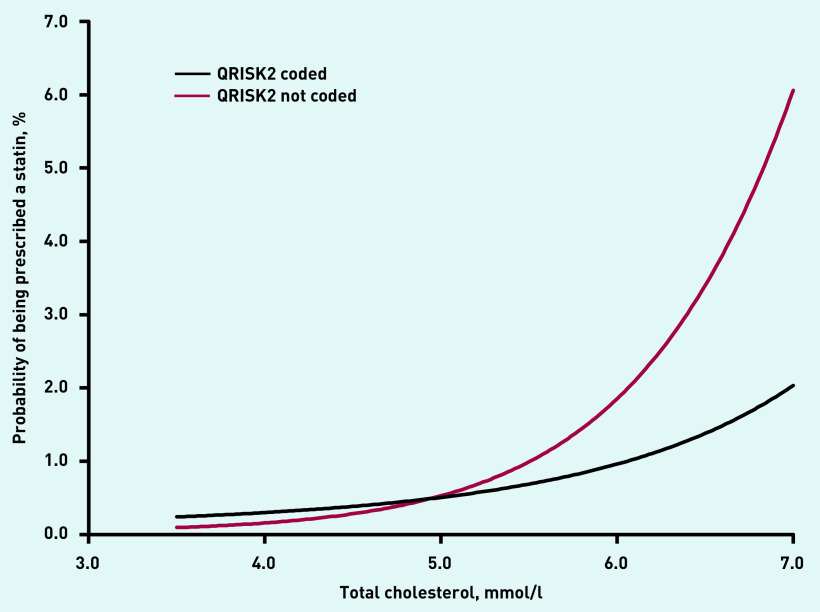
***Probability of being prescribed a statin for a theoretical patient with a QRISK2 score of 4.6% but different TC levels, shown for both QRISK2 coded or uncoded scenarios.*** ***TC = total cholesterol.***

Figure 2 illustrates the effects the TC level has on the probability of being prescribed a statin for a patient with a QRISK2 score of 4.6% in both the coded and uncoded groups. It can be seen that statin initiation is increasing likely above the standard ’upper limit of normal’ of 5.0 mmol/l but this arbitrary threshold has much less impact when a QRISK2 score is coded. The increasing probability of a statin being prescribed with increasing TC, but a constant QRISK2 score, is illustrated for two clinical scenarios in Table 2.

**Table 2.** Illustrative case

**Case 1^a^**	**Case 2^a^**
White, 50-year-old male	White, 60-year-old male
TC/HDL = 4.0	TC/HDL = 4.2
Systolic BP = 130 mmHg	Systolic BP = 155 mmHg
Non-smoker	Heavy smoker
**Actual QRISK2 score = 4.6%**	**Actual QRISK2 score = 20.3%**

	Case 1 (QRISK2 score = 4.6%)		Case 2 (QRISK2 score = 20.3%)	
	TC = 4.2 mmol/L	TC = 7.0 mmol/L	TC = 4.2 mmol/L	TC = 7.0 mmol/L
QRISK2 coded; probability of statin being prescribed, %	0.3	2.0	9.5	39.7
QRISK2 not coded; probability of statin being prescribed, %	0.1	6.1	0.7	19.3

a Year set to 2016. BP = blood pressure. HDL = high-density lipoprotein. TC = total cholesterol.

## DISCUSSION

### Summary

This study has demonstrated for the first time that coding of a QRISK2 score following a lipid test is associated with markedly different prescribing behaviour. On most occasions a QRISK2 score is not coded, but, when it is, clinicians prescribe more statins and, perhaps more importantly, prescribing is twice as likely to be consistent with guidelines. When it is coded in the record, estimated CVD risk is the strongest predictor of statin prescribing, but, when it is not, TC level and diabetic status are the strongest predictors. This supports the hypothesis that, when a QRISK2 score is not coded, clinicians are not making use of estimated CVD risk to inform their prescribing decision. If they are calculating or using clinical judgement to estimate risk then these findings suggest that their estimate is inaccurate, and determined mainly by TC level and diabetic status. The relative importance of a diagnosis of type 2 diabetes in prescribing decisions (when QRISK2 is not coded) may be a result of targets within the Quality and Outcomes Framework,^34^ which incentivised GPs to reduce TC levels in these patients.

When the QRISK2 score is low, the probability of being prescribed a statin is similar in those with and without a coded QRISK2 score and, in both cases, it is predicted by TC levels (Table 2). Measured TC level affects the probability of a statin being prescribed in both those below and above the NICE treatment threshold, indicating that TC levels influence prescribing decisions over and above their effect on QRISK2 score.

It is notable that patients in the uncoded group had considerably more entries per patient than the coded group (that is, they had their lipids measured more often before being prescribed a statin or leaving the cohort). This may be another indication of the difference in the way these patients’ GPs are managing CVD risk; perhaps repeating lipids until they get to a treatment threshold or focusing on lifestyle measures to reduce cholesterol. Further research would be required to unpick this finding.

### Strengths and limitations

This study examines a large, representative sample of general practice in England and Wales. A post-hoc QRISK2 calculation, which correlates well with coded risk estimates, enables a direct comparison of the difference in decision making between encounters where a QRISK2 score is coded and those where it is not. This helps to establish the role of risk estimation in prescribing decisions, and allows insight into the role of CVD risk on decision making when it is not coded. By choosing lipid measurement as the cohort entry criteria, it is possible to model a clinically coherent decision-making process. Additionally, using empirical data on prescribing gives a strong indication of decision-making processes while avoiding the social or professional acceptability bias that might arise if clinicians were asked to describe their decision-making processes.

In this study it is assumed that prescribing decisions were based on the data coded in the electronic patient record. It is felt that the timeframe chosen represents a clinically plausible duration where clinical decisions may be linked with the coded data. It has been assumed that, when a QRISK2 score was not coded, neither was it calculated. Although this cannot be verified, the different factors associated with prescribing between the two groups appear to corroborate this assumption. It is possible, however, that some clinicians calculated, but did not code, a risk score. Additionally, some patients may have been prescribed a statin without having their lipids measured, and therefore would not have been entered into the study cohort — it is not possible to gain insight into this decision-making process.

It is not possible to explain why patients are not started on statins, other than to state that those without a coded QRISK2 score would have been more likely to have a statin prescribed if they had a QRISK2 score coded. Patients may not have been offered statins, may have declined them, or may have decided to delay the decision to a later date.

The data used do not allow clinician-level analysis. It is possible that one of the main determinants of the use of risk estimates is clinician heuristics. It could also be that practice-level systems and culture drive prescribing decisions, as has been demonstrated in other literature.^35^ Further understanding of these influences would help guide interventions for improvement.

### Comparison with existing literature

As previously reported, a significant proportion of statin initiations are to people with low estimated CVD risk.^15^^,^^16^ The use of a CVD risk score (as indicated by the coding) was associated with an increase in, and more accurate prescribing of, statins. This is consistent with a systematic review into the impact of estimated CVD risk.^11^ Similarly, many patients with elevated CVD risk were not subsequently initiated on a statin, which is consistent with previous research.^15^^,^^16^

In contrast with other research,^21^ it was found in this study that lack of CVD risk scoring, rather than use of CVD risk scoring, was associated with overprescribing in those at low risk, and oversimplification of the decision through focusing on single risk factors. The dominance of single risk factors over global risk estimates in influencing statin prescribing has been previously reported.^18^^,^^19^^,^^36^ This focus on individual risk factors (specifically TC) is perhaps understandable, in part because patients prefer to focus on immediate problems and single risk factors rather than estimates of future risk.^23^^,^^37^

### Implications for research and practice

A significant proportion of statin initiations still seem to be based on cholesterol levels. More work needs to be done in understanding why this is and on how to change practice to focus the attention of clinicians, and patients, onto CVD risk rather than single risk factors. Risk estimation should be the cornerstone of statin initiation decisions both in deciding which patients should be offered a statin, and as a basis for shared decision making thereafter. CVD risk scoring is strongly associated with guideline-concordant prescribing. Either consultations where GPs are more likely to follow guidelines lead them to record QRISK2 scores, or recording QRISK2 scores leads GPs to be more likely to follow guidelines. If part of the explanation is the latter, then intervention to increase the recording of QRISK2 scores would increase guideline concordance.
